# Accuracy of cotinine serum test to detect the smoking habit and its association with periodontal disease in a multicenter study

**DOI:** 10.4317/medoral.21292

**Published:** 2017-06-04

**Authors:** Andrés Duque, Paula-Juliana Martínez, Astrid Giraldo, Diego F. Gualtero, Carlos-Martín Ardila, Adolfo Contreras, Silvia Duarte, Gloria-Inés Lafaurie

**Affiliations:** 1 MSc, Esp, Research Group in Basic Sciences and Clinical Dentistry, CES University, Medellin; 2 BSc, MSc, Esp, Basic-Oral Research Unit UIBO, El Bosque University, Bogota; 3 PhD, Epidemiology Group, University of Antioquia, Medellin; 4 PhD, Periodontal Medicine Group, El Valle University, Cali; 5 Esp, Dental Research Center, CIO, Pontificia Universidad Javeriana

## Abstract

**Background:**

The validity of the surveys on self-reported smoking status is often questioned because smokers underestimate cigarette use and deny the habit. It has been suggested that self-report should be accompanied by cotinine test. This report evaluates the usefulness of serum cotinine test to assess the association between smoking and periodontal status in a study with a large sample population to be used in studies with other serum markers in epidemiologic and periodontal medicine researches.

**Material and Methods:**

578 patients who were part of a multicenter study on blood biomarkers were evaluated about smoking and its relation to periodontal disease. Severity of periodontal disease was determinate using clinical attachment loss (CAL). Smoking was assessed by a questionnaire and a blood sample drawn for serum cotinine determination.

**Results:**

The optimal cut-off point for serum cotinine was 10 ng/ml. Serum cotinine showed greater association with severity of CAL than self-report for mild-moderate CAL [OR 2.03 (CI95% 1.16-3.53) vs. OR 1.08 (CI95% 0.62-1.87) ] advanced periodontitis [OR 2.36 (CI95% 1.30- 4.31) vs. OR 2.06 (CI95% 0.97-4.38) ] and extension of CAL > 3 mm [ OR 1.78 (CI95% 1.16-1.71) vs. 1.37 (CI95% 0.89-2.11)]. When the two tests were evaluated together were not shown to be better than serum cotinine test.

**Conclusions:**

Self-reported smoking and serum cotinine test ≥ 10ng/ml are accurate, complementary and more reliable methods to assess the patient’s smoking status and could be used in studies evaluating serum samples in large population and multicenter studies. Clinical Relevance: The serum cotinine level is more reliable to make associations with the patient’s periodontal status than self-report questionnaire and could be used in multicenter and periodontal medicine studies.

** Key words:**Biological markers, serum, cotinine, periodontitis, smoking.

## Introduction

Smoking is a chronic habit that causes dependence. The World Health Organization (WHO) currently estimates that each year smoking accounts for about ~6 million deaths worldwide, being the first unnatural cause of death that is associated with several diseases like aggravated cardiovascular disease and obstructive pulmonary deficiency in developed and developing countries (1). Additionally, smoking is considered one of the main risk factors in periodontitis; smokers had greater chances for more severe alveolar bone loss compared to non-smokers and responded less favorably to non-surgical periodontal therapy (2-6).

The questionnaires for self-report used to assess smoking have the advantage of being reproducible methods, non-invasive and low cost (7,8). However, there are social factors related to the patient’s overall health that can lead to a denial of the habit to avoid being stigmatized or excluded from some services health (9,10). Cotinine has been widely used as a stable biomarker of tobacco exposure and has been used to correlate its levels with periodontal disease severity (11). Although saliva cotinine test has been widely used in association studies of smoking and periodontal disease, this sample is difficult to process in multicenter studies; recently also been reported the use of serum cotinine in studies of periodontal disease in large population samples. The Serum cotinine has a longer half life, it does not require adjusting hydration difference among individuals as in saliva test (12). The saliva cotinine can be difficult to use in studies with large samples of population and multicenter studies (13,14).

In the last decades, a large number of studies have been conducted to establish the association of periodontal disease and systemic diseases (15-17) and smoking is an important confounder in this association (18,19). In many studies, serum markers are the most important test to determine these relationships. Serum cotinine can be very useful in these studies to assess smoking status for control of this confounding factor (20). A smoker absorbs half a milligram of nicotine in each cigarette, which is degraded to cotinine (the major metabolite close to nicotine), nicotine glucuronide, nircotrine and nornicotine primarily (21,22) .Nicotine possesses a very short half-life in the blood, approximately 2 h; in contrast, cotinine exhibits a longer serum half-life, approximately 19 h (23). However, serum cotinine may range from 10 to 20 ng / ml, and this variation is due to differences racial of the populations studied (24,25) .Thus determining the cutoff of cotinine in each study is important to estimate the accuracy of the surveys in detecting patients exposed vs. no exposed (26).

The aim of this study was to evaluate the accuracy of the cotinine serum test to evaluate the smoking habit and its value for detect association of smoking with severity of periodontal disease.

## Material and Methods

- Study population

The participants belonged to a multicenter study which evaluated genetic, microbiological and immunological risk factors for periodontal disease and systemic inflammatory mediators and lipid profile. The participants were previously informed about its nature, and signed a written consent previously approved by the ethics committees of the participating institutions. A total of 578 patients over 35 years of age visiting the dental clinics of five dentistry faculties including: El Bosque University and Pontificia Javeriana University, (Bogotá, Colombia), CES University and Antioquia University (Medellin, Colombia) and Valle University (Cali, Colombia) participated in the study. This report is part of a multicenter study of biological and microbiological markers, so it was necessary to exclude patients who had taken antibiotics 45 days before the sampling. The use of mouthwashes was not considered because the sample was obtained in blood. Patients with autoimmune diseases, diagnosed diabetes, taking Non-steroidal anti-inflammatory drugs (NSAIAs), pregnant or nursing women, patients undergoing orthodontic treatment and who have received periodontal treatment in the last year were excluded.

- Clinical evaluation

Medical history and clinical examination were conducted for each patient. Patients were examined by trained periodontists (AD, AG, CMA, SD). All clinical researchers underwent a calibration session on the diagnosis criteria. For the evaluation of all the risk factors of the study, CAL based case definition was used to determine the history of periodontal disease. Calibration exercise yielded an agreement ≥ 82 % for CAL. Clinical attachment levels were obtained (Intra-class correlation coefficients ICC 0.82 to 0.90) and patients were classified according to the average of the clinical attachment loss (CAL) as follows: Control patients with different degrees of gingival inflammation with a mean of CAL <2 mm at the evaluated sites; mild-moderate CAL , patients having CAL with an average of 2-4 mm; and advanced with mean of CAL > 4 mm. The extension was classified into ranges of < or ≥ 50% of affected sites with CAL >3 mm in proximal sites, but it was used in other report of the study and not in the cotinine test report. Mean of pocket depth probing (PD), bleeding on probing (BOP) and dichotomic gingival index (GI) were obtained in all patients.

- Questionnaire (self-report)

Information regarding the patient’s tobacco smoke exposure was initially collected through an interview. After one week, the smoking behavior was evaluated using a standard self-reported questionnaire. Smoking was established by any positive response by interview or self-reported questionnaire.

Sample collection and cotinine analysis: We used serum samples to assemble a cohort of patients who underwent a screening for systemic risk factor (biological markers). A blood sample was taken from each patient and serum was separated and stored at -20°C. Cotinine concentrations from the serum samples were measured using a microplate EIA**, according to the manufacturer’s instructions. To determine the concentrations of cotinine, the results of absorbance were transformed to ng/ml using a linear regression model.

- Statistical analysis: The values of sensitivity, specificity, predictive values and likehood ratios were established for the cotinine cut-off values of 10, 15 and 20 ng/ml, taking self-reported smoking as the gold standard. A ROC curve was performed within the different cotinine cut-off values. The values of sensitivity, specificity, predictive values and likehood ratios for the survey were established taking the cotinine test cut-off value as the gold standard which showed the best accuracy. To establish the association between the smoking habit evaluated by the survey and the cotinine test with the periodontal status, a Chi-square test was performed with a 5% significance level (*p* < 0.05) and OR ( odds ratio) values were calculated adjusted to geographic region, age and sex by logistic regression analysis for midle-moderate CAL and advanced CAL. Another analysis was made to the extension of CAL. For all analysis the unadjusted and adjusted models were compared using the likelihood ratio chi-square (G2), Akaike’s Information Criterion (AIC), and the Bayesian Information Criterion (BIC).

## Results

A total of 578 patients (372 women and 206 men) were studied, 209 control, 191 mild/moderate and 178 advanced ( Table 1).  Table 2 shows the Comparison of Cotinine levels with periodontal clinic parameters in patients with serum cotinine ≥10 and ≤10. There was no statistically significant association between serum cotinine cut-off and bleeding on probing and gingival index. There was a statistically significant association between serum cotinine cut-off and the mean of CAL (*p* >0,004) and PD (*p*<0,0002). In the  Table 3 and  Table 4 is presented a complete description for smokers assessed by self-report and cotinine concentrations levels (10, 15 and 20 ng/ml) , the sensitivity, specificity, predictive values and likelihood ratios for the different cotinine cut-off points of ≥ 5 ng/ml. (where self-reported smoking is the gold standard for this study). Ten nanograms of cotinine/ml in the serum showed the highest sensitivity and negative predictive value to detect non-smokers. The ROC curve confirmed a cotinine level of ≥10 ng/ml efficient to detect smokers, (0.77 the value under a curve). In a cut-off of 5 ng/ml area which analyzes the sensitivity and specificity of the diagnostic test decreases according to the ROC curve (0.76), for this reason analysis is not required below the cutoff of 10 ng / ml. A cotinine cut-off point ≥ 10 ng/ml detected the highest percentage of smoking patients. 118 participants (20.4%) referred being smokers in the self-report, while 127 participants (22%) were positive for the serum cotinine test at cut-off point level 10 ng/ml. When both methods were combined, i.e. a positive result for either the questionnaire or the serum cotinine test, identified 166 patients as smokers (28.7%) ( Table 5).

**Table 1:**
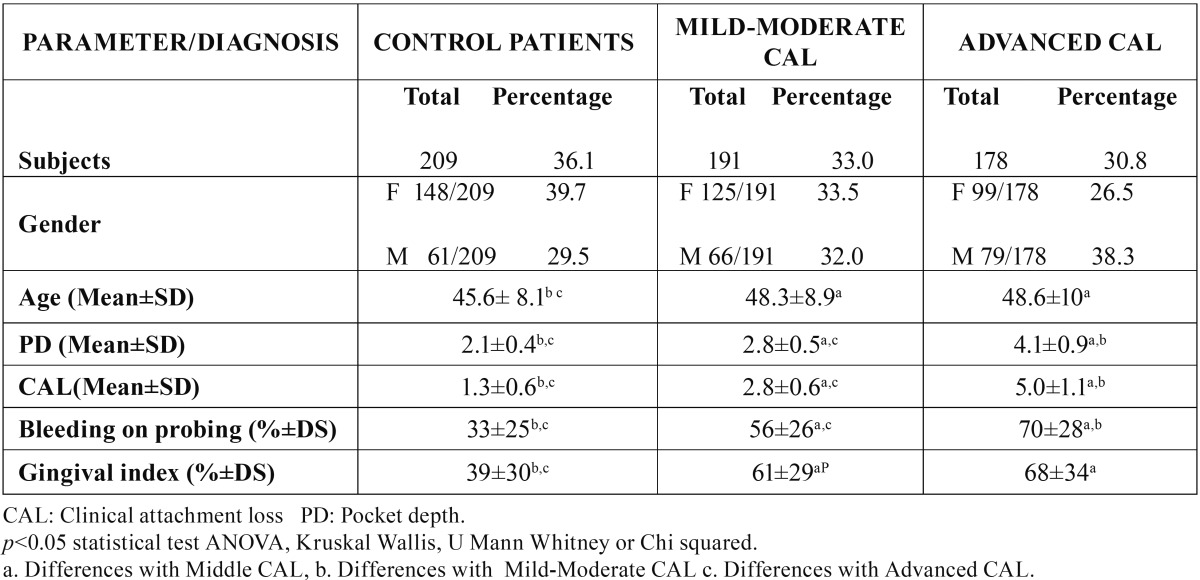
Demographic characteristics of the population evaluated and clinical parameters of periodontal status.

**Table 2:**
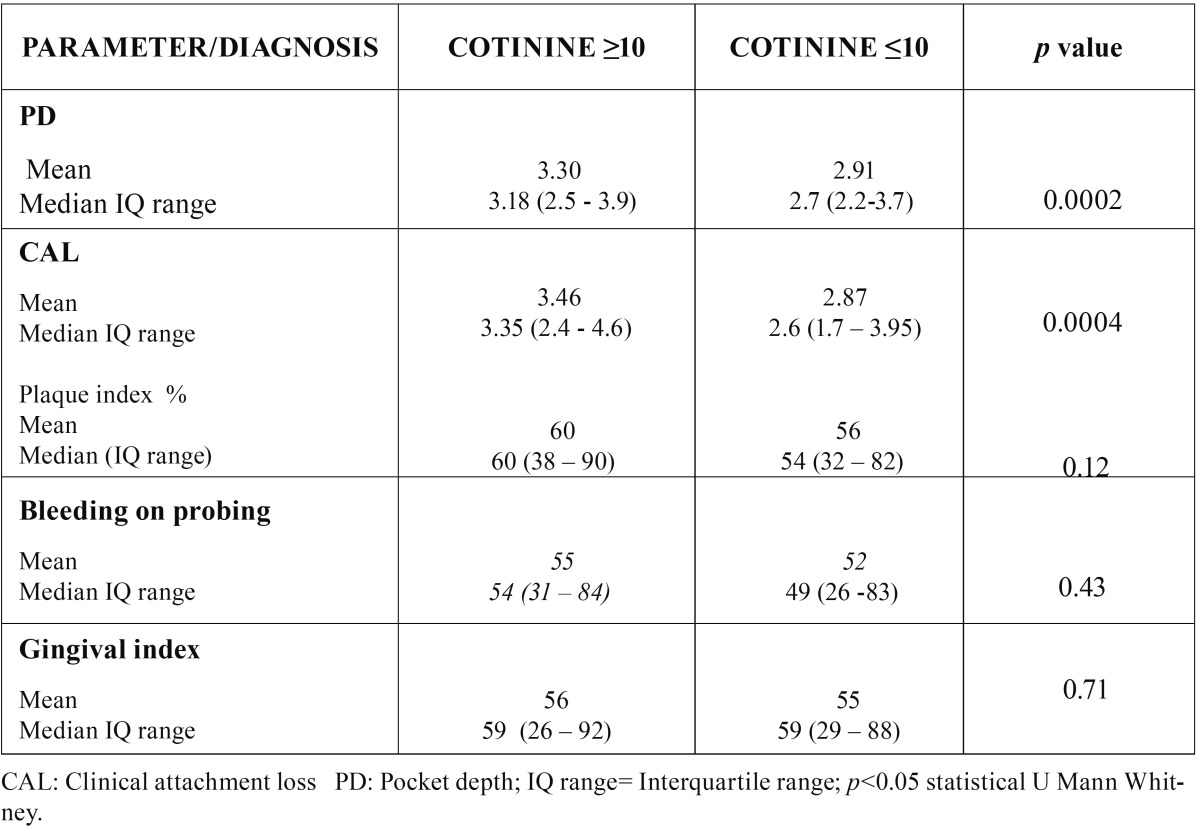
Comparison of Cotinine levels with periodontal clinic parameters.

**Table 3:**
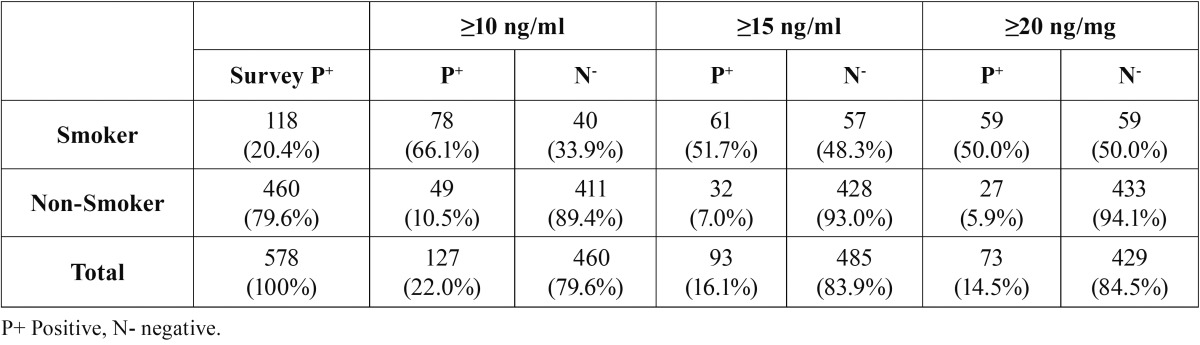
Distribution of smokers and non-smokers determined self-reported smoking and serum cotinine test.

**Table 4:**
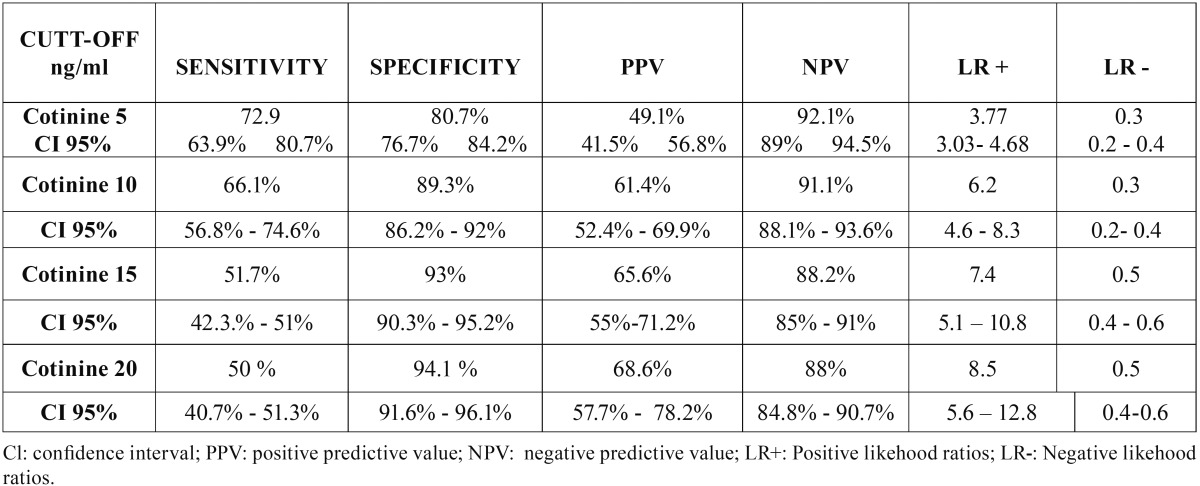
Accuracy of serum cotinine cut-off points of 10 ng/ml aimed to detect smokers using data collected by survey as the gold standard.

**Table 5:**
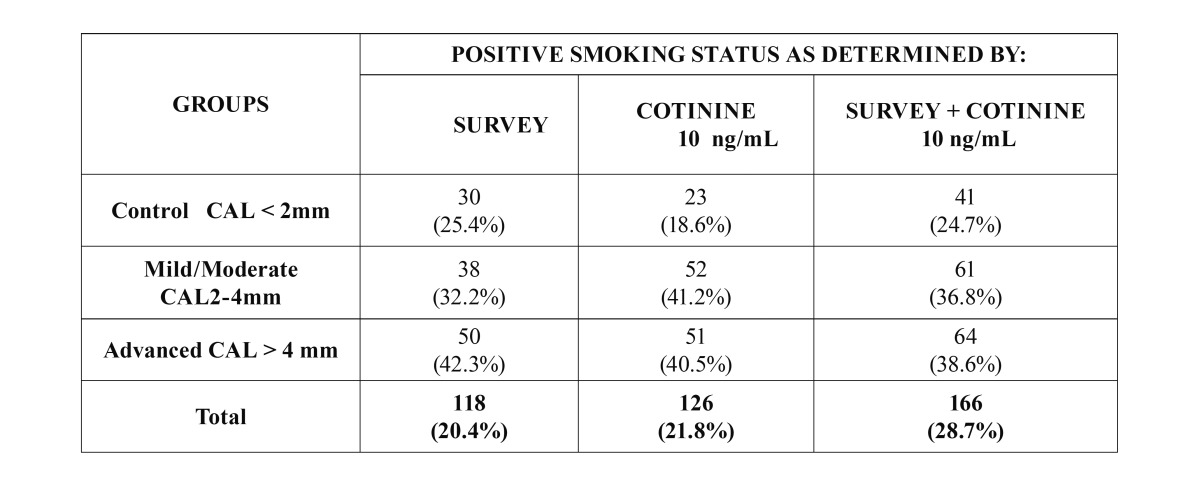
Prevalence of smokers according to patient’s periodontal status evaluated by survey and/or 10 ng/ml serum cotinine levels.

The  Table 6 shows a significant association between smoking and mild- moderate CAL when evaluated by cotinine levels ≥10 ng/ml (OR 2.03 (CI95% 1.16-3.53)) but these association was lost when was evaluated by either the survey (OR 1.08 (CI95% 0.62-1.87)). In severe periodontitis serum cotinine test (≥10 ng/m) shows greater association (OR 2.36 (CI95% 1.30-4.31)) than self-report questionnaire (OR 1.80 (CI95% 1.01-3.22)). When the two tests were evaluated together was not shown to be better than serum cotinine test (OR 1.76 CI 95% 1.04-3.00)) for advanced CAL.

**Table 6:**
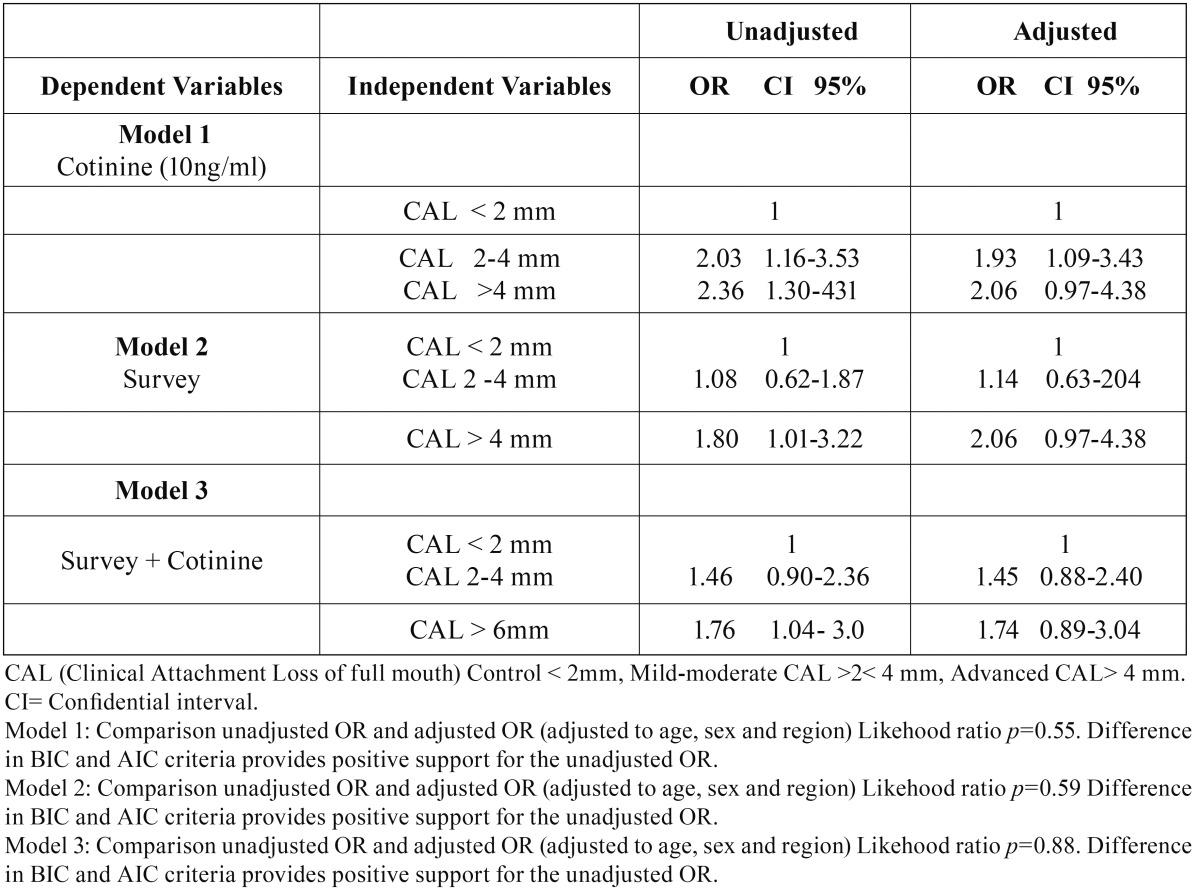
Association of cigarette smoking with severity of clinical attachment loss evaluated by the test of cotinine, the survey and both methods.

## Discussion

Cotinine is the major metabolite of nicotine and the biomarker of choice because it is not influenced by diet or the environment surrounding the patients, thus fulfilling requirements of specificity and half-life in the body (27). Levels from 10 to 20 ng/ ml due to variability in diverse racial groups (24,25), and uncut-off of 10ng/ml was the most sensitivity for detect smoking habit in the population evaluated in this study.

The identification of environmental exposure to tobacco is difficult to determine through surveys. This is troubling since there is evidence about the risk of periodontitis in passive smokers (13). Complement the smoking habit survey with cotinine test could be useful to detect passive smokers or those who deny the habit. Serum cotinine test shows more association with periodontal status evaluated by clinical attachment level loss and pocket depth than smoking habit self-report questionnaires. This evidence seems to support the use of serum cotinine test in studies with a large population where serum samples are obtained. Serum cotinine had been utilized in epidemiologic studies in cardiovascular disease and other systemic diseases (28).

Although saliva cotinine test has been widely used in association studies of smoking and periodontal disease, this sample is difficult to process in multicenter studies; recently also been reported the use of serum cotinine in studies of periodontal disease in large population samples (29,30).

Among current smokers, there was a dose-response relationship between cigarettes smoked per day and the odds of periodontitis (31). There is strong evidence that smokers have more CAL and periodontal pockets (32,33). These findings are in agreement with the results obtained in this study given that the patient’s smoking active status correlated with the severity of periodontal disease. There is strong evidence of the association of smoking and severe periodontitis evaluate by CAL and PD; Current smoking is a significant predictor of clinical attachment loss in longitudinal studies. However, in this study the association between smoking evaluate by cotinine with bleeding on probing and gingival index were not significant; others studies had shown that smoking exerts a dose-dependent suppressive effect on gingival bleeding on probing (34). A hypothesis about these findings is that there were positive patients to cotinine but they were passive smokers. In addition the cut-off point of 10 ng/ml may not be determinant to demonstrate changes in the BOP.

In general, both the smoking survey and the serum cotinine tests independently showed associations with the presence and severity of periodontal disease. This study also explored the association between the primary study variable (CAL based case definition) and the severity of periodontitis. Cotinine levels at a cut-off of 10 showed a greater association with the severity of the periodontal disease evaluated by CAL. Cotinine test may be influenced by the time between the last exposure and sampling of blood (35). In this study 35% of patients surveyed reported smoking test were negative in cotinine; 75% smoked less than 10 cigarettes per day and 40% showed levels <10 ng / ml of cotinine in serum (data not shown). People that do not smoke frequently and occasional smokers could have negative test since cotinine is not detected 30 hours after smoking cessation (36). Although serum cotinine test did not identify some patients at the cutoff point of 10 ng/ml, it was more sensible to establish associations with periodontal status possibly because identify patients with more active habit. Interestingly, 13.1% of the patients who reported non-smoke in the survey were positive in the serum cut-off point of 10 ng/ ml cotinine test; patients in the questionnaires can hide or lie about smoking by social fears.

The serum cotinine test has limitations such as the impossibility to determine the time of smoking exposure, number of cigarettes smoked per day, and both methods can be complementary in the evaluation of smoking habit when these variables are relevant in the study.

## Conclusions

Self-reported smoking and serum cotinine test ≥ 10ng/ml are accurate and complementary methods to assess the patient’s smoking status. The cotinine level is more reliable to make associations with the patient’s periodontal status. Serum cotinine could be used in studies evaluating serum samples in large population and multicenter studies.
